# What Should Be the Focus of Treatment When Insomnia Disorder Is Comorbid with Depression or Anxiety Disorder?

**DOI:** 10.3390/jcm12051975

**Published:** 2023-03-02

**Authors:** Charles M. Morin, Suzanne M. Bertisch, Rafael Pelayo, Nathaniel F. Watson, John W. Winkelman, Phyllis C. Zee, Andrew D. Krystal

**Affiliations:** 1 Department of Psychology, Brain Research Centre, Laval University, Quebec, QC G1V 0A6, Canada; 2 Department of Medicine, Brigham and Women’s Hospital, Harvard Medical School, Boston, MA 02115, USA; 3 Department of Psychiatry and Behavioral Sciences, Stanford University Sleep Medicine Center, Redwood City, CA 94305, USA; 4 Department of Neurology, University of Washington School of Medicine, Seattle, WA 98195, USA; 5 Department of Psychiatry and Neurology, Massachusetts General Hospital, Boston, MA 02114, USA; 6 Department of Neurology, Center for Circadian and Sleep Medicine, Northwestern University, Evanston, IL 60611, USA; 7 Departments of Psychiatry and Neurology, UCSF Weill Institute for Neurosciences, San Francisco, CA 94158, USA

**Keywords:** sleep disturbances, treatment, pharmacological, cognitive-behavioral therapy comorbidity, psychiatric, depression, anxiety, clinical appraisal

## Abstract

Insomnia is a significant, highly prevalent, persistent public health problem but often remains undiagnosed and untreated. Current treatment practices are not always evidence-based. When insomnia is comorbid with anxiety or depression, treatment often targets that comorbid condition with the expectation that improvement of the mental health condition will generalize to sleep symptoms. An expert panel of seven members conducted a clinical appraisal of the literature regarding the treatment of insomnia when comorbid anxiety or depression are also present. The clinical appraisal consisted of the review, presentation, and assessment of current published evidence as it relates to the panel’s predetermined clinical focus statement, “*Whenever chronic insomnia is associated with another condition, such as anxiety or depression, that psychiatric condition should be the only focus of treatment as the insomnia is most likely a symptom of the condition*”. The results from an electronic national survey of US-based practicing physicians, psychiatrists, and sleep (N = 508) revealed that >40% of physicians agree “at least somewhat” that treatment of comorbid insomnia should focus solely on the psychiatric condition. Whereas 100% of the expert panel disagreed with the statement. Thus, an important gap exists between current clinical practices and evidence-based guidelines and more awareness is needed so that insomnia is treated distinctly from comorbid anxiety and depression.

## 1. Introduction

Insomnia is characterized by both nighttime and daytime symptoms involving dissatisfaction with sleep duration or quality and difficulties falling asleep, problems staying asleep, and early morning awakenings. Those sleep problems are associated with daytime impairments such as fatigue, difficulties with concentration and sustained attention, and mood disturbances [1]. 

Insomnia is highly prevalent, with about 30% of adults reporting insomnia symptoms and 10% meeting the criteria for an insomnia disorder [2]. Insomnia is more common among women, older adults, individuals with poor physical or mental health, and those with atypical sleep schedules [2]. Not only is insomnia highly prevalent, but it is also frequently a persistent condition, with persistence rates of 70.7%, 49.4%, and 37.5% over 1-, 3-, and 5-year follow-up periods, respectively [3]. 

While insomnia produces an immediate negative impact on quality of life, when persistent, it may exacerbate pre-existing symptoms and also increase the risk for new onset or recurrence of previous psychiatric conditions, especially depression and anxiety [4]. Several longitudinal studies have documented this risk; individuals with persistent insomnia present a two-fold increase in developing depression over the next few years compared to those whose insomnia remits or is treated with either CBTI or pharmacological intervention during the same interval [5]. While this association has been reported more often for major depression, additional studies have also documented a link between prior insomnia and a moderately increased risk for other psychiatric conditions such as depression, anxiety, alcohol abuse, and psychosis [6,7]. Other studies have shown that insomnia is often comorbid with depression or anxiety with rates of comorbidity near 50% [8]. 

When patients present in clinical practice with both insomnia and anxiety or depression, clinicians have been inclined to treat the psychiatric condition and not directly target the sleep problem, assuming the latter would remit upon adequate treatment of the psychiatric problem. Such practice may have resulted from insomnia being conceptualized as a symptom of another (primary) disorder, typically depression or anxiety disorders [9]. This dichotomization of insomnia as primary or secondary has led many clinicians to focus on treating the so-called primary problem and assume that it would also improve sleep [10,11]. However, much evidence published in the last two decades has shown that insomnia may either precede or follow the onset of psychiatric disorders and given this bidirectional relationship, patients may benefit from treatments that directly target insomnia even in the context of comorbid mood disorders [12,13]. Such evidence has led to a shift in paradigm, most notably in the Diagnostic and Statistical Manual of Mental Disorders-5th edition (DSM-5) and the International Classification of Sleep Disorders 3rd edition (ICSD-3), i.e., insomnia is recognized as a disorder on its own and may necessitate direct treatment even when another psychiatric disorder is comorbid [1,14]. Despite this important shift in paradigm since the publication of DSM-5 and ICSD-3, many clinicians may still consider insomnia as a symptom of another disorder, namely depression or anxiety, and continue to focus treatment on the psychiatric disorder. 

Given the importance of defining best insomnia treatment practices based on current evidence, a clinical appraisal regarding whether to treat insomnia distinctly from any potential comorbidities was conducted to evaluate the validity of the following predetermined clinical focus statement: “*Whenever chronic insomnia is associated with another condition, such as anxiety or depression, that psychiatric condition should be the only focus of treatment as the insomnia is most likely a symptom of the condition*”. The primary goal of this clinical appraisal was to highlight discrepancies between evidence-based best practice regarding the focus statement and the self-reported assumptions concerning best practice of practicing physicians in the field. The appraisal consisted of three parts:

1. A national survey, consisting of seven statements regarding clinically related insomnia topics and including our focus statement, to understand current U.S. physician perspectives regarding insomnia treatments.
2. A review of the current literature related to the treatment of insomnia where comorbid anxiety and depression are present.
3. A meeting of the expert panel in their role as insomnia experts to assess the evidence, the strength of the literature presented and its implications for insomnia treatment and to identify clinical research that will enable improved outcomes.

## 2. Materials and Methods

### 2.1. National Survey

Prior to the clinical appraisal meeting, an electronic survey was distributed nationally to US-based practitioners (Doctor-MD, Doctor-PhD, Nurse Practitioners and Physician Assistants). The survey sought to understand their opinions on a number of distinct but related issues regarding insomnia management that are the subject of current debate in the medical community. These issues were crafted into predetermined clinical focus statements to be voted on as part of the national survey. The survey was distributed by email to two distinct practitioner cohorts, in two waves: December 2021 and April 2022. 

In Wave 1: the online survey was sent to 97,000 HCPs procured from a variety of collector lists and all emails used were General Data Protection Regulation compliant. A total of 155 responses were collected, and 47 respondents were removed as not qualified via demographic questions such as What is your area of specialty? Additionally, what percentage of your time is spent insomnia management? The datapoint of 100 respondents was agreed to be the threshold for legitimacy for publications of this type, but given the low response rate of 0.2%, a second wave was launched in April 2022. The dataset requirements for Wave 2 were the same as Wave 1 and the survey was sent to an additional set of 70,319 HCPs. With a response rate of 0.6%, a further 400 eligible respondents were collected. With both waves only those healthcare providers who spend at least 10% of their time managing insomnia patients were included in the analysis. In total, n = 508 eligible responses to the statement that is the subject of this report were obtained and of the 508 total the breakdown subspecialty is as follows: MDs (74%), nurse practitioners (14%), physician assistants (11%) and PhD (1%).

Practitioners involved in insomnia patient management (from specialties including PCPs/Family practice, psychiatrists, and sleep specialists) were asked to indicate their level of agreement with each survey statement. The levels were as follows:

1. Strongly agree.
2. Mostly agree, but with minor reservations.
3. Slightly agree, but with major reservations.
4. Slightly disagree, due to minor reservations.
5. Mostly disagree, due to major reservations.
6. Strongly disagree.

Respondents also had the opportunity to provide written comments regarding each statement and >95% of them provided additional comments regarding the rationale behind their level of agreement with the focus statement. 

### 2.2. Expert Panel and Literature Review

The statement, “Whenever chronic insomnia is associated with another condition, such as anxiety or depression, that psychiatric condition should be the only focus of treatment as the insomnia is most likely a symptom of the condition” was evaluated during a December 2021 meeting of the expert panel. One of the panel members (CMM) was tasked with the presentation of an unbiased review of the current literature pertaining to this statement, presenting both statement-supporting and statement-refuting evidence. The purpose of the meeting was to undertake a balanced and unbiased discussion of the evidence, with a focus on evaluating the validity of this statement as it applies to sleep medicine. The expert panel also assessed how the evidence relates to current perceptions, as demonstrated by the national survey results.

A literature search was performed in October 2021 using PubMed and the Cochrane Database with the following search terms shown below in Table 1 with limits set for inclusion/exclusion:

Exclusions based on observational study, low participant number, no control group, or involved an insomnia medication without FDA approved indication for treatment for insomnia were applied. Of the initial 87 results for depression and 53 results for anxiety, 7 unique studies were selected for presentation to the expert panel. The additional search that was limited to article type meta-analysis produced 1 additional article that was selected for inclusion.

### 2.3. Nature of the Evidence

Prior to presentation of the selected studies, the expert panel anonymously indicated their level of agreement with the appraisal statement using the same six-point scale provided to national survey respondents. Following presentation of the evidence, the expert panel rated the quality of the evidence according to the following criteria:

1. Evidence obtained from meta-analysis, including at least one large, randomized controlled trial (RCT).
2. Evidence obtained from either meta-analysis, including at least one small RCT, or from at least one well-designed large RCT.
3. Evidence obtained from well-designed cohort or case–control studies.
4. Evidence obtained from case series, case reports, or flawed clinical trials.
5. Opinions of respected authorities based on clinical experience, descriptive studies, or reports of expert committees.
6. Insufficient evidence to form an opinion.

Following presentation of the literature, the expert panel indicated their level of agreement with the appraisal statement a second time, noting whether the presented evidence affected their assessment. The expert panel discussed how the evidence related to current insomnia treatment management and practice and identified future needs.

## 3. Results

### 3.1. Survey Results

**Figure 1:**
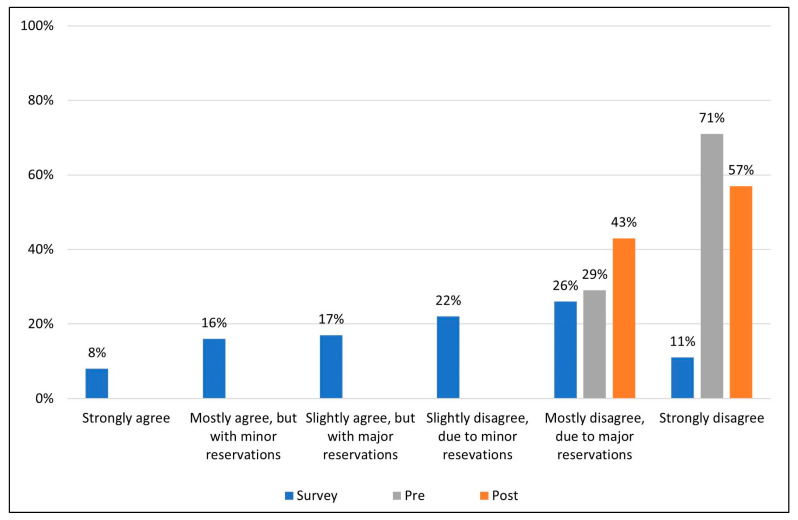
Level of acceptance/rejection for field survey respondents and the expert panel pre- and post-presentation of evidence. The 508 survey respondents voted on their level of acceptance or rejection of the statement based on a 6-point Likert scale (blue). Prior to seeing field survey results, and discussing the literature, the 7 members of the appraisal panel voted on their level of acceptance/rejection (gray). The literature and data supporting and refuting the statement were then reviewed, discussed and the same 7-member panel voted once more (orange) on their levels of acceptance/rejection using the same 6-point Likert scale.

**Figure 2:**
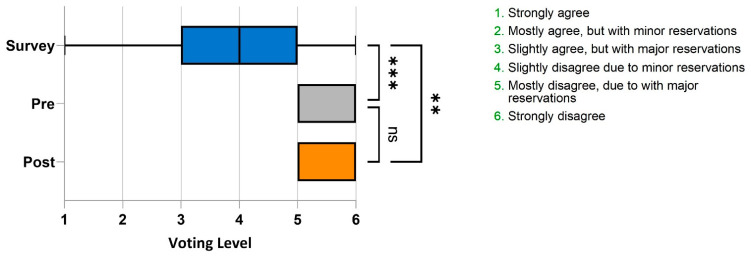
Median voting level for National Survey participants (blue bar, n = 508) and Panelists pre- (grey bar, n = 7) and post-presentation (orange bar, n = 7) for the statement “*Whenever chronic insomnia is associated with another condition, such as anxiety or depression, that psychiatric condition should be the only focus of treatment as the insomnia is most likely a symptom of the condition*”. The grading on the x-axis corresponds to the levels of support/rejection of the statement, with interquartile ranges and anova *p* values as shown. *** denotes a significant a statistically significant *p* value of 0.0007; ** denotes a significant *p* value of 0.0017. ns denotes a non-significant difference in values.

74% of eligible national survey respondents were MDs, while the rest were PhDs, NPs or PAs. Breakdown by specialty was as follows: Family Medicine 56.0%, Internal medicine 29.5%, Psychiatry/Psychology 9.8%, Sleep specialist 0.8%, Other 3.9%. The national survey demonstrated considerable variability in the level of support for the statement, “*Whenever chronic insomnia is associated with another condition, such as anxiety or depression, that psychiatric condition should be the only focus of treatment as the insomnia is most likely a symptom of the condition*” among the respondent practitioners (Figure 1), reflecting the complexity of current thinking on this issue. Survey respondents (n = 508) expressed a range of opinions that included all six levels of support for the statement, though the majority (59%) expressed some level of disagreement with the statement. Furthermore, the median level of support across all national survey respondents, using the 1–6 scale, with 1 = strongly agree to 6 = strongly disagree was 4 indicating some disagreement with the statement (Figure 2). Nevertheless, 41% of the survey respondents at least slightly agreed with the statement.

Based on the respondents’ comments, disagreement tended to be due to the knowledge that insomnia, depression, and anxiety are all significant issues in treating patients. Each needs to be considered individually as well as collectively in understanding and treating patients. Some sleep specialists noted that insomnia in some cases is the primary cause of their problems and some family care practitioners commented that only focusing on one aspect is a flaw in many treatment plans. The survey respondents that agreed with the statement took the approach that insomnia was the symptom, “not the etiology” and often would opt to treat what they believed to be the underlying issue first and only address the insomnia if it did not go away with the treatment of anxiety or depression.

During the December 2021 clinical appraisal meeting, expert panelists indicated their level of agreement with the statement “Whenever chronic insomnia is associated with another condition, such as anxiety or depression, that psychiatric condition should be the only focus of treatment as the insomnia is most likely a symptom of the condition” both prior to and following presentation of the evidence discussed in this review. 

The pre-presentation panel survey resulted in all of the seven experts expressing mostly disagreement due to major reservations (level 5), or strongly disagree (level 6) (Figure 1). This trend remained during post-presentation voting. In Figure 2, the median vote level for panelists was 6, both pre- and post-presentation, and the mean panelist voting level, was 5.7 ± 0.18 (SEM) and 5.57 ± 0.2 (SEM) prior to and following the presentation. The median field survey vote was 4, with a mean of 3.75 ± 0.07 (SEM), with survey respondents in general only mildly disagreeing with the statement. This represents a significant difference in voting levels between survey responders and panelists both pre-presentation (*p* = 0.0007) and post-presentation of the evidence (*p* = 0.0017).

These results strongly suggest that while the panel of sleep specialists, both before and after presentation of the clinical evidence, understand that insomnia should always be treated as a separate co-morbid disorder, a sizeable proportion of prescribing non-insomnia specialists do not share this understanding, and continue to treat comorbid insomnia as a symptom of anxiety and/or depression.

### 3.2. Literature Review

Insomnia is a risk factor for several psychiatric conditions. Breslau et al. investigated the association between sleep disturbance and psychiatric disorders cross-sectionally and prospectively and showed that insomnia was a significant predictor of subsequent major depression [6]. More recently, Hertenstein et al. carried out a systematic review and meta-analysis to investigate whether persistent insomnia is a risk factor for future psychopathology [7]. A meta-analysis of 13 prospective studies using a diagnostic-based insomnia definition and a minimum follow-up of 12 months showed that insomnia was a significant predictor of depression (OR 2.83, 95% CI 1.55–5.17) and anxiety (OR 3.23, 95% CI 1.52–6.85), even when using only the 11 studies with no mental disorder at baseline

Belleville et al. explored the efficacy of cognitive behavioral therapy (CBT) for insomnia and CBT for generalized anxiety disorder (GAD) in a small cohort of women with comorbid GAD and insomnia [15]. Using a single-case methodology, 10 women (mean age = 45 y/o) with chronic insomnia and GAD were randomly assigned to 1 of 2 groups: (1) CBT for GAD followed by CBT for insomnia, or (2) CBT for insomnia followed by CBT for GAD. Sleep and anxiety were measured via diagnostic interviews, daily diaries, and self-report questionnaires. Time series analyses revealed significant improvements on anxiety, worry, and sleep after CBT for GAD. Following CBT for insomnia, significant changes were observed on sleep but, to a lesser extent, on anxiety and worry measures. The researchers concluded that although this study is based on a very small sample, in the presence of comorbid GAD and insomnia, initiating treatment for GAD first produced superior clinical benefits in both anxiety and sleep, compared to a treatment that focused first on insomnia.

A further study on CBT-I for patients with comorbid insomnia and depression compared CBT-I + antidepressant medication (escitalopram) against treatments that target solely insomnia (CBT-I + placebo) or solely depression (escitalopram + sleep hygiene control) [16]. The randomized clinical trial took place in two urban academic clinical centers, with participants (n = 107 adults; 68% female, mean age 42 ± 11) diagnosed with MDD and insomnia. All groups reported sleeping better after treatment, with large effect sizes, but no significant group differences. On polysomnography measures, only the CBT-I groups improved, while sleep worsened among patients treated with the antidepressant escitalopram. All groups improved on insomnia and depression symptoms severity, including those that were only treated for insomnia or only for depression. Although those findings are equivocal, this study provides some evidence that we should treat the co-existing depression to improve insomnia.

Conversely, a randomized control trial performed by Manber et al. showed that CBT-I enhances depression outcome in patients with comorbid major depressive disorder and insomnia [17]. The sample size was small (30 adults; 61% female; mean age 48.6 ± 13 with comorbid insomnia and depression) but they were able to show the benefit of adding CBT-I to the antidepressant medication escitalopram, achieving higher remission rates for both depression and insomnia among patients receiving CBT-I. This study gives further support to the idea that sleep needs to improve if we expect depression to remit. Blom et al. carried out a three-year follow-up study comparing CBT for depression to CBT for insomnia, for patients with both diagnoses [18]. Insomnia treatment was more effective (*p* < 0.05) than depression treatment to reduce insomnia severity and equally effective in reducing depression severity. The researchers concluded that patients with comorbid insomnia and depression should be offered treatment for insomnia, in addition to treatment for depression.

The meta-analysis conducted to derive the AASM practice guidelines on the psychological and behavioral therapies of insomnia provided a strong endorsement recommendation that multi-component CBT-I (without concurrent medication) was effective for insomnia either with or without comorbid psychiatric disorders [19].

There are also several studies that have looked at adding insomnia medications for the treatment of comorbid insomnia and depression. A large study of 545 adults (~age 41 y/o) was conducted to evaluate the effect of adding eszopiclone treatment for insomnia to fluoxetine treatment for depression. Such co-therapy produced significant improvements on sleep latency, wake after sleep onset, and total sleep time, as well as greater reductions of depressive symptoms compared with fluoxetine plus placebo [20]. A randomized control trial was carried out by the same group of researchers to investigate whether zolpidem extended release (ER), co-administered with the anti-depressant escitalopram, improved sleep in comorbid insomnia and depression [21]. A total of 385 adults with comorbid insomnia and depression were treated with escitalopram 10 mg/d. Half of the patients received concomitant zolpidem ER 12.5/n and half received placebo for 8 weeks. Patients treated for both insomnia and depression showed greater improvement in insomnia and next-day functioning compared to patients treated with the anti-depressant alone. A similar study was also undertaken to investigate the effects of zolpidem extended release, co-administered with escitalopram, to improve sleep in comorbid insomnia and GAD patients [22]. A large cohort of 383 adults, aged 21–64 y/o, with comorbid insomnia and GAD were treated with escitalopram 10 mg/d; half received additional zolpidem ER 12.5/n and half received placebo for 8 weeks. Patients treated for both anxiety and insomnia improved more than those treated only for anxiety on most sleep outcomes.

More recently, a randomized clinical trial was conducted to investigate the effectiveness of sequential psychological and medication therapies for insomnia disorder [3]. This involved 211 patients (132 women; mean [SD] age, 45.6 [14.9] years) with a chronic insomnia disorder, including 74 patients with a comorbid anxiety or mood disorder were randomly assigned to first-stage therapy involving either behavioral therapy (BT; n  =  104) or zolpidem (zolpidem; n  =  107); those who did not remit received a second treatment involving medication (zolpidem or trazodone) or psychological therapy (BT or Cognitive Therapy; CT). The primary end points were the treatment response and remission rates, defined by the Insomnia Severity Index total score. Focusing on the subset of patients with comorbid mood or anxiety disorder, their overall response and remission rates after initial therapy were lower compared to patients without psychiatric comorbidity. However, among those patients with psychiatric disorders, the addition of a second stage therapy was more beneficial when that second stage therapy involved CT or trazodone, two treatments that targeted mood symptoms as opposed to focusing exclusively on sleep, as with BT and zolpidem. Thus, those findings suggest that treatment of patients with comorbid insomnia may need to target not only sleep but also focus on mood. 

### 3.3. Quality and Impact of Evidence

Overall, all seven experts assessed the presented evidence to be of high quality (level 2—evidence from a meta-analysis or RCTs). Similar to the pre-presentation vote, the post-presentation panel vote revealed a strong disagreement with the statement, “Whenever chronic insomnia is associated with another condition, such as anxiety or depression, that psychiatric condition should be the only focus of treatment as the insomnia is most likely a symptom of the condition”. Overall, 4 of the 7 experts strongly disagreed with the statement (level 6), and the remaining 3 experts expressed mostly disagreement due to major reservations (level 5) (Figure 2). The median level of support for the statement post-presentation, on the 1–6 scale, did not differ from 6 pre-presentation to 6 post-presentation.

## 4. Discussion and Conclusions

In the context of insomnia treatment, the current literature provides strong evidence against the statement, “Whenever chronic insomnia is associated with another condition, such as anxiety or depression, that psychiatric condition should be the only focus of treatment as the insomnia is most likely a symptom of the condition”.

Most of the research evidence summarized in this presentation did not fully support the statement. In general, studies showed that a treatment that focused solely on depression or anxiety produced some benefits on sleep/insomnia, but those improvements were significantly less than those obtained with co-modal interventions targeting both sleep and anxiety or depression [17,18,20,21,22]. Two publications using CBT-I were identified as somewhat supportive of the statement in that treatment of anxiety/depression produced better outcomes on insomnia and psychological outcomes than treatment focusing on insomnia [15,16].

A rare study that looked at possible mechanisms [17] of action suggested that sleep improvements may mediate reduced depressive symptoms. Previous research has shown that sleep disturbance is the most common residual symptom among patients treated for depression [23] and persistence of such symptoms among otherwise remitted patients increase the risk of relapse for depression [24]. Together those findings lend additional support to the need of treating sleep disturbances even in a context of comorbidity with another psychiatric disorder such as major depression. Therefore, even if the benefit is more modest for some patients, it may still be clinically meaningful to add a specific insomnia therapy. 

An important issue that was not directly addressed in the reviewed literature regards the best timing or sequencing of treatments. Should both conditions be treated concurrently or sequentially? Although no specific study directly examined that question, the only study that used sequential therapies for insomnia disorder (including some patients with comorbid anxiety/depression) found that when a second stage therapy is necessary, it is more effective if it is a treatment with a broader target than just sleep [3]. Patients with comorbid insomnia and treated with behavioral therapy or zolpidem as first-stage therapy had a better final outcome when the second stage therapy included either cognitive therapy or trazodone, two therapies that are not specific to sleep but may also have an antianxiety or antidepressant effect.

The clinical manifestation of insomnia is frequently comorbid with psychiatric disorders, particularly anxiety and depression, with co-occurrence in at least 40–50% of adults from the general population [6,8]. Based on older paradigms, insomnia has long been conceptualized as symptomatic of other medical or psychiatric conditions and, accordingly, treatment was essentially symptomatic [9]. The belief and underlying practice were that by treating the underlying (primary) condition, insomnia would remit once the underlying condition was successfully treated. Despite a significant shift in that paradigm since the advent of the revised classification of sleep disorders about a decade ago, in which insomnia was recognized as a disorder in its own right with the implication that it should be targeted for treatment even when it is comorbid with another condition, one must recognize that for a substantial proportion of clinicians, perceptions/beliefs about best treatment practices have not shifted to align with the current treatment paradigm or evidence [1,14].

The results from the survey of physicians are both informative and surprising. That more than 40% of practicing physicians agree “at least somewhat” with the statement that treatment of comorbid insomnia should focus solely on the psychiatric condition suggests an important gap between current clinical practices and evidence-based guidelines. The main implication of this finding is the need to expand education initiatives designed to raise awareness of the bi-directional relationship between insomnia and mental health and, more importantly, strengthen the dissemination of training programs on insomnia management.

## Figures and Tables

**Table 1:** Literature search method with keywords, limits and exclusion criteria.

Keywords	Limits	Abstracts Reviewed	Exclusion Criteria	Publications Selected for Presentation
“Insomnia comorbidities AND depression”	date limits set for inclusion from 2011–2021 and limited to clinical trials or randomized control trials	87	Observational study, low participant number, no control group, or involved an insomnia medication without FDA approved indication for treatment for insomnia	5
“Insomnia comorbidities AND anxiety”	date limits set for inclusion from 2011–2021 and limited to clinical trials or randomized control trials	53	Observational study, low participant number, no control group, or involved an insomnia medication without FDA approved indication for treatment for insomnia	3
“Insomnia comorbidities AND depression”	date limits set for inclusion from 2011–2021 and limited to meta-analysis	23	Observational study, low participant number, no control group, or involved an insomnia medication without FDA approved indication for treatment for insomnia	1
“Insomnia comorbidities AND anxiety”	date limits set for inclusion from 2011–2021 and limited to meta-analysis	18	Observational study, low participant number, no control group, or involved an insomnia medication without FDA approved indication for treatment for insomnia	1

## Data Availability

Not applicable.
